# Exploring Health Literacy and Vascular Access Decision Making: A Scoping Review

**DOI:** 10.3390/jcm13133734

**Published:** 2024-06-26

**Authors:** Shayna Brathwaite, Olamide Alabi, Lynne Simpson, Nader Massarweh

**Affiliations:** 1 Surgical and Perioperative Care, Atlanta VA Healthcare System, Decatur, GA 30033, USA; olamide.alabi@emory.edu (O.A.); nader.nabile.massarweh@emory.edu (N.M.); 2 Division of Vascular Surgery, Department of Surgery, Morehouse School of Medicine, Atlanta, GA 30310, USA; 3 Division of Vascular Surgery and Endovascular Therapy, Department of Surgery, Emory University School of Medicine, Atlanta, GA 30322, USA; 4 Department of Surgery, Morehouse School of Medicine, Atlanta, GA 30310, USA; 5 Information Services, Morehouse School of Medicine, Atlanta, GA 30310, USA; lsimpson@msm.edu; 6 Division of Surgical Oncology, Department of Surgery, Emory University School of Medicine, Atlanta, GA 30322, USA

**Keywords:** end stage kidney disease, hemodialysis, health literacy, vascular access, knowledge

## Abstract

One in seven adults in the United States has chronic kidney disease (CKD) and individuals with the most severe form, end stage kidney disease (ESKD), may require renal replacement therapy with hemodialysis. Despite well-established guidelines indicating that arteriovenous access is the preferred type of vascular access for hemodialysis, in 2021, 85.4% of patients initiated dialysis with a CVC. While the reasons for this evidence–practice gap are unclear, health literacy and patient disease-specific knowledge may play an important role. Importantly, 25% of patients with CKD have limited health literacy. While there is an abundance of research regarding the presence of poor health literacy, poor kidney disease-specific knowledge, and their association with health outcomes in patients with CKD, there is currently a paucity of data about the relationship between health literacy, vascular access-specific knowledge, and vascular access outcomes. The aim of this narrative review is to describe the relationship between health literacy, disease-specific knowledge, and vascular access in patients with CKD. A better understanding of health literacy in this population will help inform the development of strategies to assess patient vascular access-specific knowledge and aid in vascular access decision making.

## 1. Introduction

One in seven adults in the United States has chronic kidney disease (CKD) and they account for over USD 86 billion in Medicare expenditure [1,2]. Over 90% of individuals with CKD and 30% of those with stage 4 CKD (i.e., those with ‘advanced CKD’ who are nearing kidney failure) do not even know they have this condition [1]. The prevalence of CKD is highest amongst Black individuals, those aged 65 years and older, and those with lower income as well as lower educational attainment [1,2]. The clinical manifestations of CKD can range from no symptoms at all (typically among people with mild CKD) to fatigue, muscle aches, swelling, and shortness of breath in those with the most severe form of CKD—end stage kidney disease (ESKD). Individuals with ESKD require renal replacement therapy (RRT). In 2021, 59.9% of patients requiring RRT utilized hemodialysis, 8.3% utilized peritoneal dialysis, and 31.8% underwent kidney transplantation [2]. ESKD is associated with a significant decrease in life expectancy, with the 5-year survival for newly diagnosed patients being less than 50% [2].

Individuals with ESKD face complex decisions alongside their healthcare providers and caretakers including, but not limited to, the timing of dialysis initiation, the mode of dialysis (i.e., hemodialysis vs. peritoneal dialysis), and for those on hemodialysis, the best option for the vascular access site (e.g., distal vs. proximal upper extremity) as well as the type of vascular access (e.g., arteriovenous access vs. central venous catheter [CVC) [3]. Despite well-established guidelines indicating arteriovenous access is the preferred type of vascular access for hemodialysis, in 2021, 85.4% of patients initiated dialysis with a CVC and 23.0% of the total population on dialysis at that time had a CVC in place [2,3,4,5]. CVCs are associated with a number of adverse clinical outcomes including central venous steno-occlusive disease, infection, and increased all-cause and cardiovascular mortality [5]. While there may be limitations in the type of vascular access a patient is a candidate for, due to multiple factors such as anatomic considerations or patient life expectancy that limit the utility of arteriovenous access, we believe that there are also patient-, provider-, and system-level factors that contribute to the evidence–practice gap. Specifically, patient and provider factors may include patient disease-specific knowledge, health literacy, and patient–provider communication. Patient–provider communication challenges are highlighted by the fact that even patients who have regular visits with their healthcare providers report a poor understanding and insufficient knowledge of their disease to make decisions regarding vascular access [6,7,8,9,10].

Health literacy is defined as ‘the ability to access, understand, appraise, and apply basic health information to make decisions regarding their healthcare’ [11,12]. Health literacy is necessary to make decisions that promote disease prevention as well as maintain and/or improve quality of life [13]. Approximately 80 million adults in the United States are described as having limited health literacy, with those who are other than White race, those with lower socioeconomic status, and older adults disproportionately accounting for these individuals [13,14,15]. Low or limited health literacy is associated with poor disease-specific knowledge and comprehension which leads to difficulty navigating the healthcare system and can lead to poor health-related outcomes [14]. For example, individuals with limited health literacy are more likely to have poor medication adherence, are less likely to utilize preventative health services, more frequently visit the emergency room, experience readmissions at higher rates, and have higher rates of mortality [15,16,17,18,19]. Health literacy is the product of multiple sociodemographic factors such as culture, language, income, educational attainment, and situational determinants such as familial and social support [12]. More recent definitions of health literacy by the Centers for Disease Control include an individual’s ability as well as an organization’s ability to equitably enable patients to find, understand, and use the information and available services [11]. This highlights the importance of patient trust in the healthcare system and patient–provider communication as an integral part of health literacy [12,20]. Health literacy and effective communication between patients and the healthcare team are particularly important when managing chronic and complex health conditions, such as CKD.

Approximately 25% of patients with CKD have limited health literacy. Limited health literacy has been defined as a score of 0–22 on the Short Test of Functional Health Literacy in Adults (STOFHLA) and a score of 0–60 on the Rapid Estimate of Adult Health Literacy in Medicine (REALM) questionnaires [19]. As demonstrated in previous studies, patients with low or limited health literacy may be more likely to initiate dialysis using a CVC. There is a critical need to improve the understanding and knowledge about decisions regarding vascular access among patients with CKD to improve the utilization of arteriovenous access rather than a CVC. Vascular access decision making impacts quality of life and overall prognosis in patients with ESKD [21]. As such, patients must have a reasonable understanding of their medical condition and the factors that affect these aforementioned decisions in order to fully participate in discussions with their providers. Such complex healthcare decision making can be particularly challenging for those individuals with limited disease-specific knowledge and limited health literacy. Because complex healthcare decision making can be particularly challenging for individuals with limited disease-specific knowledge and limited health literacy, and because decisions regarding vascular access can have an important impact on ESKD patients’ quality of life and overall prognosis, there is a critical need to improve patients’ understanding and knowledge of CKD and ESKD to enhance their comfort and ability to participate in shared decision making. While there is an abundance of research regarding the presence of poor health literacy, poor kidney disease-specific knowledge, and their association with health outcomes in patients with CKD, there is currently a paucity of data about the relationship between health literacy, vascular access-specific knowledge, and vascular access outcomes [19]. The aim of this narrative review is to describe the relationship between health literacy, disease-specific knowledge, and vascular access in patients with CKD. A better understanding of health literacy in this population will help inform the development of strategies to assess patient health literacy and vascular access-specific knowledge, and aid in vascular access decision making.

## 2. Materials and Methods

**Figure 1:**
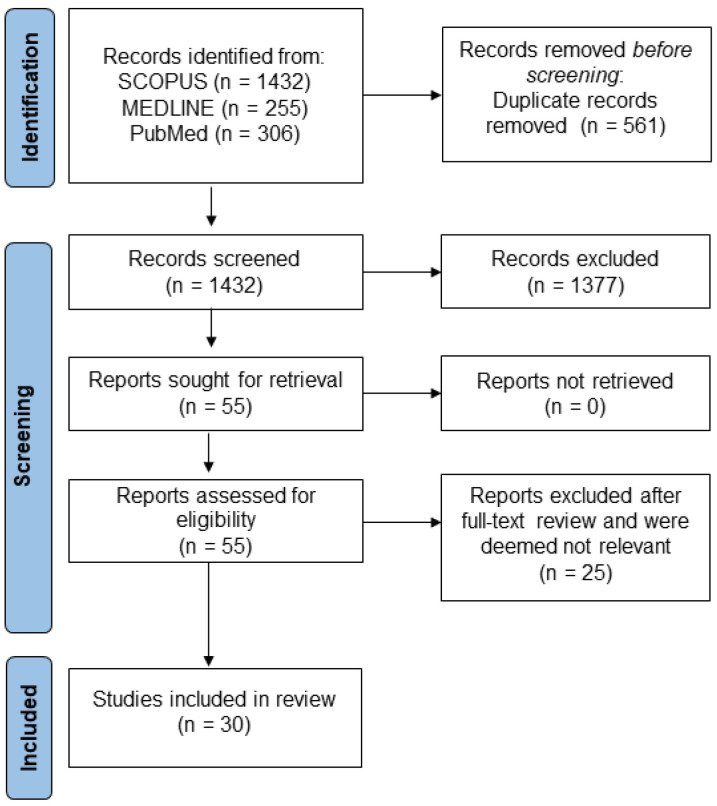
PRISMA flowchart of study selection process.

This scoping review has been reported in accordance with the PRISMA extension for scoping reviews. A literature search was performed using Scopus, PubMed, and MEDLINE databases to identify articles published between 1 January 2000 and 31 January 2023. Only full-text publications and those published in the English language were included. Search terms were generated around three main concepts: ‘Chronic Kidney Disease’, ‘End Stage Renal Disease’, or ‘End Stage Kidney Disease’; ‘Vascular Access’; and ‘Health Literacy’ or ‘Knowledge’. The search terms are listed in Table 1. Additional studies were identified manually through a review of the references in the studies identified in our literature search. The initial search yielded 1432 results and included journal articles, review articles, meta-analyses, and clinical trials. Abstracts were reviewed by one author (SB). After review, publications were excluded if they were not related to the topic of interest and/or did not include information about health literacy, disease-specific knowledge, shared decision making, chronic kidney disease, or end stage kidney disease. After these initial exclusions, 55 full-text manuscripts were identified and, after review of the full text of each, an additional 25 publications were excluded as they were not related to the topic of interest. During the review of full-text publications, the reason for exclusion was recorded. In total, 30 articles were used to inform this review. The flow of articles from identification to final inclusion is represented in Figure 1.

## 3. Results

In the Paasche-Orlow model of health literacy, the continuum from health literacy to patient outcome is impacted by three distinct domains: (1) patient access to and utilization of the healthcare system; (2) patient self-care; and (3) patient–provider interactions [22]. Patient-level factors such as self-efficacy, perceived barriers to health service navigation, patient disease-specific knowledge, participation in decision making, and health education all play a role in patient outcomes [22]. The Paasche-Orlow health literacy framework can be applied to patients with CKD to understand how limited health literacy may be associated with poor vascular access-specific outcomes, at the patient, provider, and system level. During our literature search, we ensured that all distinct domains of the Paasche-Orlow model were represented, and the model was also used to frame our analysis. As such, the findings from our literature review are presented in the context of this model. 

### 3.1. Health Literacy in CKD

A significant proportion of patients with CKD and, more specifically, those with ESKD on dialysis have limited health literacy [7,19]. In a systematic review, Taylor et al. found that the pooled prevalence of limited health literacy in non-dialysis-dependent CKD patients was 25%, 27% in those with ESKD, and 14% in kidney transplant patients [7]. For patients in the earlier stages of CKD, low health literacy has been associated with more rapid progression of disease [23]. Patients with low health literacy may delay seeking care due to a poor understanding of clinical signs that warrant them seeking care [22]. Additionally, patients with limited health literacy have an overall discomfort in healthcare social interactions which can further limit their engagement in preventative care services for CKD [22]. Additionally, in patients with CKD, demographic factors that are independently associated with limited health literacy include self-identifying as a race other than White and markers of low socioeconomic status, such as having less than a high school education and an income of less than USD 30,000 per year [7,24]. Among patients with CKD, limited health literacy is also associated with poor self-management behaviors including poor medication adherence, missed dialysis treatments, and low adherence rates to dietary and fluid recommendations [24,25]. In fact, compared to ESKD patients with adequate health literacy (as measured by the Rapid Estimate of Adult Health Literacy in Medicine tool), ESKD patients with limited health literacy have a higher risk of death after adjusting for common risk factors such as age, sex, race, and diabetes mellitus [3,16]. Given the high prevalence of poor health literacy in patients with CKD, health literacy may represent an important potential target for national quality improvement efforts focused on enhancing shared decision making, optimizing care pathways, and improving clinical outcomes related to vascular access.

#### 3.1.1. Healthcare Access and Utilization and Vascular Access Decision Making

For patients with advanced CKD or ESKD, decision making regarding vascular access can be complicated. For example, the pathway to vascular access includes pre-dialysis care by a nephrologist, shared decision making with healthcare providers in selecting a dialysis modality, as well as discussions regarding the risks and benefits of hemodialysis (as compared to peritoneal dialysis) and selecting the type of vascular access (arteriovenous access versus central venous catheter). After shared decision making with providers and caregivers, patients are then referred to a vascular access surgeon before initiation of dialysis or soon thereafter. If patients are deemed to be appropriate candidates for vascular access, they subsequently undergo surgery for arteriovenous vascular access placement.

Several strategies have been utilized to improve arteriovenous vascular access placement, including the use of care coordinators and educational programs for patients and/or providers. While employing a care coordinator with the goal of streamlining care for patients with ESKD is associated with less frequent hospitalizations, it unfortunately does not appear to increase the utilization of arteriovenous access [26]. The fact that care coordination and the streamlining of care do not lead to improved outcomes suggests that there is persistent care fragmentation and poor communication between patients and providers [26]. In contrast, the involvement of a dialysis access coordinator increases the utilization of arteriovenous vascular access by 9.4%, and the involvement of a vascular access care coordinator improves arteriovenous access use through overall coordination of surgical waiting lists by ensuring that surgical priority for the creation of vascular access goes to patients with the greatest need [27,28,29]. Additional interventions associated with modest improvements in the utilization of arteriovenous access include the implementation of a guideline-directed program for providers and educational programs for patients to discuss treatment options [30]. Perhaps unsurprisingly, patients with the greatest ability to engage with healthcare providers, to navigate the healthcare system, to identify good health information from reliable sources, and to understand health information well enough to know what to do with it had a higher prevalence of arteriovenous vascular access relative to CVC use [6]. These findings suggest that higher health literacy and the ability to navigate the healthcare system improve utilization of arteriovenous vascular access.

#### 3.1.2. Determinants of Central Venous Catheter Utilization

Patients with poor health literacy may have lower disease-specific knowledge which in turn may impact their ability to self-manage their disease. Better self-management behaviors, such as consumption of a healthy diet, participation in physical activity, medication adherence, and avoidance of nephrotoxins, are more commonly found in patients with higher objective and perceived knowledge of their kidney disease. While patients with higher objective or perceived kidney disease knowledge have higher rates of CKD self-care behaviors such as smoking avoidance, medication adherence, and adherence to diabetic care routines, CKD self-care behaviors do not appear to differ based on health literacy [31]. By comparison, lower perceived kidney disease knowledge is associated with lower health literacy, lower income, lower educational attainment, and importantly, poor satisfaction with provider communication [32]. Similarly, higher perceived (but not objective) kidney disease knowledge is associated with improved self-care knowledge. Patient perception also impacts the quality of life of patients with CKD. Generally, patients with low health literacy and CKD have lower physical and mental healthcare quality of life [33]. Compared to patients with a CVC, patients with arteriovenous access have improved quality of life scores and improved perception of their current therapy [34]. Despite improved quality of life with arteriovenous access, up to 60% of patients may refuse early arteriovenous access placement, and of those who refuse, 23.3% report that the reason is poor understanding of their disease and vascular access options [35,36]. Overall, the current literature suggests that, independent of objective knowledge or health literacy, patient confidence and perception are important factors contributing to quality of life and self-care management [31,37].

#### 3.1.3. Patient and Provider Relationships and Vascular Access Decision Making

1. Building the trust relationship

Effective communication between patients and their providers is an essential component of improved health literacy and outcomes in patients with CKD and ESKD. When patients develop a trusting relationship with their providers, they are more likely to accept provider recommendations and to feel as though they are participants in the decision making related to their health [38,39]. Cassidy et al. highlighted the importance of a patient-centered framework for patient–provider interactions when dealing with CKD and found that effective communication and greater patient engagement can overcome barriers associated with limited health literacy and education [40,41]. In patients with advanced stage CKD, the primary factors influencing decision making regarding the choice of dialysis modality are receiving education tailored to the individual, the availability of educational resources (e.g., printed educational material or CKD-specific websites), and adequate time allocated for healthcare provider(s) to educate and provide information to patients [42]. Unfortunately, many patients remain stuck in the early decision making stages regarding their choice of renal replacement therapy because they are waiting on clear, expert guidance from their healthcare providers to inform their treatment decisions [41]. It is important to note that more frequent communication with healthcare providers and higher healthcare utilization do not improve patient self-efficacy or perceived kidney disease knowledge [32]. Even though more than 50% of patients with CKD see their kidney doctors at least three times per year, 25% report not knowing why they were sent to see a kidney specialist [32]. To foster the trusting relationship and the quality of communication between patient and provider, patients have suggested that providers spend more time with them and that they thoroughly discuss any recommendations and follow up with patient progress, particularly after any major health events [38]. Additionally, communicating with empathy and avoiding paternalistic communication styles may improve the quality of communication [39]. Together, these factors suggest that in addition to health literacy, improving the quality and content, not just the frequency, of patient–provider interactions may potentially improve patient kidney disease knowledge and enhance their ability to make care decisions.

2. Improving kidney disease-specific knowledge

Patients having greater trust in their providers could improve the effectiveness of educational programming because patients feel more confident that their providers are giving them the best information. When receiving effective educational programming and having robust patient–provider communication, patients with both high and low health literacy have improved disease-specific knowledge and healthcare behaviors and outcomes [43]. In a study of 2274 new dialysis patients, limited health literacy alone was not associated with increased hemodialysis catheter usage [10]. Similarly, limited health literacy alone is not an independent predictor of the type of vascular access selected and used [17]. Educational programs such as the Kidney Early Evaluation Program (KEEP) or the National Treatment Options Program (NTOP), which were designed to improve ESKD patient knowledge and preparation for dialysis, have significantly increased kidney transplantation and the use of peritoneal dialysis and are associated with longer survival after dialysis initiation [30,44]. However, these programs have inconsistent results regarding improved use of arteriovenous access rather than a CVC for dialysis [30,44]. Patients initiating dialysis who participated in the NTOP education program were 2.14 times more likely to initiate dialysis with arteriovenous access, while those who participated in KEEP did not have higher rates of permanent arteriovenous access placement [30,44]. The fact that participation in education programming consistently improves outcomes for patients initiating dialysis, such as rates of transplantation and mortality, but does not consistently and significantly improve vascular access outcomes suggests that there is something unique about vascular access outcomes and decision making. Additionally, the fact that an educational program alone does not improve arteriovenous access use may be due to the fact that a patient receiving educational programming at a single time point might not be sufficient to affect vascular access decision making. An ideal education program for patients regarding vascular access should be continuous, multidisciplinary, and structured such that it is tailored to an individual patient’s goals and preferences [45,46]. Importantly, when education programming is effective, improves patient emotional preparedness, and increases patients’ knowledge regarding dialysis, patients are more likely to initiate dialysis with arteriovenous access rather than a CVC and continue to use arteriovenous access moving forward [47].

3. Collaborative decision making

The types of collaborative decision making between patients and healthcare providers that are typically associated with a greater use of arteriovenous vascular access (rather than a CVC) include discussions about prognosis, flexibility in decision making approaches (e.g., consideration of patients’ goals and preferences as well as adapting vascular access to suit the individual), and recurring opportunities to revisit decisions regarding management with providers [48]. Shared decision making about vascular access requires the provider to present the pros and cons of vascular access, elicit patient and caregiver values and perspectives, provide opportunities to ask clarifying questions, and make recommendations that are respectful of the clinical context and individual patient preferences [49,50]. Interestingly, despite receiving pre-dialysis renal replacement counseling and education, many patients indicate that they lack sufficient knowledge or clarity to make a decision regarding the use of a CVC or arteriovenous access [37,51]. The vast majority of patients (84%) report that their physician’s recommendation is an important influencing factor when making a choice about vascular access modality [52]. This highlights the importance of a trusting patient–provider relationship. In a qualitative study of 96 patients with advanced CKD, interactions and communication with healthcare providers were routinely identified as factors affecting dialysis and vascular access decision making, and trust in their providers overrode previous fears regarding dialysis or vascular access [37]. Additional considerations that patients reported during vascular access decision making included fears regarding dialysis initiation, practical concerns such as pain, cost, burdening caregivers, and lifestyle disruptions, all of which can potentially be mitigated by discussion and collaborative decision making between patient and provider [37].

Similarly, multiple studies have found that greater objective health-related knowledge was associated with greater arteriovenous access use by patients [3,52]. Individuals with lower knowledge scores on the Chronic Hemodialysis Knowledge Survey (CHeKS) were more likely to use a CVC at initiation of dialysis compared to arteriovenous access. Additionally, at 6 months after dialysis initiation, patients in the highest CHeKS quintiles had a higher proportion of arteriovenous access as compared to those in the lowest quintile [53]. Again, this suggests that improving patient education regarding kidney disease and vascular access, which are components of a patient’s health literacy, could in turn improve vascular access outcomes.

### 3.2. Limitations

During a review of the literature about the association between health literacy, vascular access-specific knowledge, and vascular access outcomes, we noted that there were no studies that addressed this topic in its entirety. Therefore, we performed a scoping review to understand the landscape of the topic of interest, but there are inherent limitations in performing a broad scoping review. There is significant heterogeneity of the literature in terms of the study type, included participants, and findings such that we cannot make generalizable conclusions from this study. Although this scoping review cannot provide conclusions about the association between CKD and ESKD patient knowledge and vascular access outcomes, it does provide an exploration of the current literature as a tool to identify gaps in the knowledge and develop possible interventions. A secondary limitation of this work is that only one author performed a critical appraisal of all included studies. This fact does lead to a potential for reviewer bias, but this was mitigated by keeping our search broad and including all of the relevant literature.

## 4. Discussion

This review suggests that there may be a correlation between health literacy, disease-specific knowledge, and vascular access outcomes, although the correlation is not well understood. When specifically considering CKD and ESKD outcomes, such as quality of life, medication adherence, and the utilization of healthcare systems, the data are clear and abundant that health literacy, in addition to other patient, provider, and system characteristics, plays a significant role. Given the gap in the literature regarding health literacy and vascular access outcomes, it is imperative to also explore these associations in future studies. Given the benefits of arteriovenous dialysis access in patients with CKD, it is necessary to identify tools that can increase utilization of this type of dialysis vascular access. The onus for this shift is not only on the patient to acquire and integrate information into their decision making, but also for providers to improve and develop trusted communication with patients and share tailored education that patients can understand. Improved education and communication may lead to improved patient understanding of the risks and benefits of arteriovenous access and in turn higher rates of utilization of this modality. Existing tools for measuring kidney disease-specific knowledge appear adequate, but patients with CKD may benefit from the creation of instruments to measure vascular access-specific knowledge more accurately. It also appears that developing focused educational programming as well as improving the quality of the information provided by providers (which may include primary care physicians, nephrologists, vascular surgeons, and other vascular access interventionalists) may improve patient knowledge and self-care and aid in vascular access decision making. In conclusion, there is a need to measure patient vascular access knowledge and health literacy to better understand their role in vascular access decision making.

## Figures and Tables

**Table 1:** Narrative review search terms.

Search Terms
“kidney disease” or “renal disease” or dialysis or hemodialysis or “renal replacement” AND “health literacy” or education or “decision making” or retention or knowledge AND Arteriovenous or fistula or AVF or graft or AVG or “vascular access” or “central venous catheter” or CVC or catheter or “central venous line” or CVL or permcath or permacath AND “health care” or healthcare or communication or “decision making” or “health behavior” AND “Chronic Kidney disease” or “end stage kidney disease” or “end stage renal disease” or “dialysis knowledge” or “health literacy” or “vascular access” or “dialysis access”

## Data Availability

No new data were created or analyzed in this study. Data sharing is not applicable to this article.
